# Adult Sellar Region Atypical Teratoid/Rhabdoid Tumor: A Retrospective Study and Literature Review

**DOI:** 10.3389/fneur.2020.604612

**Published:** 2020-12-15

**Authors:** Fujun Liu, Shucai Fan, Xin Tang, Shuangmin Fan, Liangxue Zhou

**Affiliations:** Department of Neurosurgery, West China Hospital, Sichuan University, Chengdu, China

**Keywords:** atypical teratoid/rhabdoid tumor, clinicopathologic and radiologic features, adult female, sella region, treatment

## Abstract

**Background:** Adult sellar region atypical teratoid/rhabdoid tumor (AT/RT) is a rare lesion. We aimed to elucidate clinical, radiologic, and pathological characteristics, treatment strategies, and outcomes of this disease.

**Methods:** Five adult sellar AT/RT patients were retrospectively analyzed between January 2015 and December 2018. In addition, we performed a review of the reported data on adult sellar AT/RT.

**Results:** Patients (*n* = 5) were female with a median age of 50 years. The mean duration of symptoms, of which headache was the most frequent, was 1.6 months (range, 2 weeks−8 months). The average tumor size was 2.82 cm (range, 1.9–4.5 cm). All lesions were irregularly shaped. MRI showed heterogeneous enhancement in three of five lesions. Four of five patients underwent subtotal resection (STR) and one gross total resection (GTR). Whereas, one patient received post-operative adjuvant radiotherapy, one patient received post-operative combination of radio- and chemotherapy. The review of the reported data showed that 39 cases of adult sellar AT/RT had been reported. The estimated median overall survival (OS) was 23 months with a 1-year survival estimate of 59.7%. The median OS for patients with GTR was 28 months and 17 months for patients with STR. Kaplan–Meier analysis showed that patients with high (≥35%) MIB-1/Ki67 index value had a significantly shorter OS compared with those with low (<35%) index value (*p* = 0.033), and that patients who received post-operative combination radio- and chemotherapy had longer OS than that of those who did not (*p* < 0.001).

**Conclusion:** Adult sellar region AT/RT is a rapidly growing tumor with a poor prognosis. High levels of MIB1/Ki-67 on histology may indicate aggressive feature of the tumor. Maximal safe resection followed by adjuvant radiotherapy combined with chemotherapy may be the optimal therapeutic strategy for adult sellar region AT/RT.

## Introduction

Atypical teratoid/rhabdoid tumor (AT/RT) of the central nervous system (CNS), which occurs chiefly in children below the age of 3 years, is an aggressive malignant tumor (1, 2). According to the 2016 WHO Classification of Tumors of the CNS, AT/RT is defined as having specific genetic alterations in the *INI1/SMARCB1/hSNF5*, or rarely, in the *SMARCA4/BRG1* genes (3). Initially described in the early 1990s by Horn et al. (4), adult AT/RT is an extremely rare tumor, commonly located in the sellar region (5), with a lifetime risk estimated at <1 per million individuals (6). A recent study of AT/RT showed hemangiopericytoma-like stag-horn vasculature and lack of INI1 expression in the adult sellar region featured by rhabdoid tumors, which was identified as a clinicopathologically and genetically distinct variant of AT/RT (7). However, the clinical and radiological features, pathological characteristics, treatment strategies, and clinical outcomes of adult sellar region AT/RT remain ill-defined due to its rarity. In the present study, we report the clinical features, radiologic findings, pathological characteristics, treatment, and outcomes of a series of five adult sellar region AT/RT diagnosed in our hospital and also review the literature related to adult sellar region AT/RT found on the PubMed database and Google Scholar.

## Methods

### Patients

Between January 2015 and December 2018, five adult patients with sellar region AT/RT received surgical resection in the neurosurgery department of West China Hospital of Sichuan University. The diagnosis of the adult sellar region AT/RT was based on the 2016 WHO Classification of CNS Tumors (3). This study was approved by the West China Hospital Ethics Committee. Written informed consent had been obtained from all patients' families previously.

### Clinical Data

Relevant data were extracted retrospectively from patients' medical and surgical records. Clinical data including patient age, sex, symptoms, duration of symptoms before diagnosis, tumor size, extent of surgical resection (subtotal resection or gross total resection), endocrine hormone levels, histological findings, surgical outcomes, and clinical follow-up were analyzed.

All patients had pre-operative gadolinium-enhanced cranial MRI scans, including T1-weighted, T2-weighted, and gadolinium-enhanced T1-weighted sequences. The size of the tumors, cystic lesions, characteristics of contrast enhancement, peri-tumor edema, tumor shape, and hydrocephalus were obtained from the pre-operative post-contrast imaging. The tumor size in MRI was defined as the largest diameter of the lesion. The characteristics of contrast enhancement of MRI were classified into homogeneous and heterogeneous contrast enhancement. CT was performed to help with pre-operative differential diagnosis and CT density, calcifications, and intratumoral hemorrhage were also recorded. Tumor specimens were fixed in 10% buffered neutral formalin, paraffin-embedded, sectioned, and processed for H&E staining for routine histological evaluation and immunohistochemical staining.

The extent of surgical resection was classified into gross total resection (GTR; no distinct residual tumor) and subtotal resection (STR; >90% of tumor removal), which was evaluated according to the surgeon's operative notes and <72 h post-operative imaging analyses. Patients were assessed by an oncologist for adjuvant therapy after surgical resection. The post-operative adjuvant radiotherapy, chemotherapy, and overall survival (OS), defined as the time from date of surgical resection until death or last follow-up, were reviewed. The neurologic functional outcomes were also assessed using the Karnofsky performance scale (KPS). The endpoint of this study was the OS and recurrence-free survival (RFS); RFS was defined as the time between surgical resection and tumor recurrence on neuroimaging studies.

### Statistical Analysis

IBM SPSS Statistics (version 24, IBM Corp.) were applied for data analysis. For quantitative data, means ± SD or median are presented. Categorical data are presented as frequencies and percentages. OS was analyzed using Kaplan–Meier estimates and the log-rank test for comparisons. A *p*-value ≤0.05 was considered as statistically significant.

### Systemic Analysis

PubMed and Google Scholar were searched for case reports and series relevant to adult sellar AT/RT reported up to October 12, 2020. “Sellar,” “Suprasellar,” “Adult,” and “Atypical Teratoid/Rhabdoid Tumor” were used as either key words or medical subject headings. In addition, references from articles previously identified were used for further cases. The results were manually screened for studies focusing on clinical experience in diagnosis and/or treatment of adult sellar AT/RT in human subjects.

## Results

### Patient Characteristics

Of the five patients in our institution, all were female, and median age at diagnosis was 50 years (range, 29–80 years). The most common symptom was headache (*n* = 5; 100%), which was usually severe and unbearable, followed by visual disturbance (*n* = 3; 60%), ptosis (*n* = 2; 40%), numbness in limbs (*n* = 2; 40%), and galactorrhea (*n* = 1; 20%). The average duration of symptoms before surgery was 1.6 months (range, 2 weeks−8 months). The examination of cerebrospinal fluid for five patients was normal on admission to hospital. The pre-operative hormone profiles including luteinizing hormone (LH), prolactin, growth hormone (GH), cortisol, plasma adrenocorticotropic hormone (ACTH), follicle-stimulating hormone (FSH), thyroid-stimulating hormone (TSH), and free thyroxine 4 (FT4) are listed in Table 1. Four patients had hypopituitarism and one patient had a normal anterior pituitary function.

**Table 1:** Pre-operative levels of various hormones in 5 adult patients with sellar region AT/RT.

**Case number**	**FSH (mIU/ml)**	**LH (mIU/L)**	**Prolactin (ng/ml) (6.0–29.9)**	**GH (ng/ml) (0.126–9.88)**	**ACTH (ng/L) (5.0–78)**	**Cortisol (nmol/L)**	**TSH (mU/L) (0.27–4.2)**	**FT4 (pmol/L) (12.0–22.0)**
1	6.7	0.8	29.54	1.03	15.68	480.79	1.25	9.93
2	**4.1**	0.39	**120.28**	2.51	5.998	**59.15**	0.38	**7.15**
3	**1**	0.1	**1.92**	**0.12**	**1.62**	**40.13**	0.548	**7.31**
4	4.03	0.34	7.86	0.98	34.31	**17.02**	0.368	**4.55**
5	**0.7**	**<0.1**	20.08	0.26	**2.59**	403.5	1.9	**4.04**

*FSH, follicle-stimulating hormone (follicular phase: 3.5–12.5 mIU/ml; ovulatory phase: 4.7–21.5 mIU/ml; luteal phase: 1.7–7.7 mIU/ml; menopause: 25.8–13.8 mIU/ml); LH, luteinizing hormone (follicular phase: 2.4–12.6 mIU/ml; ovulatory phase: 14.0–95.6 mIU/ml; luteal phase: 1.0–11.4 mIU/ml; menopause: 7.7–58.8 mIU/ml); GH, growth hormone; ACTH, adrenocorticotropic hormone; TSH, thyroid-stimulating hormone; FT4, free thyroxine 4. Bold means abnormal values*.

### Radiologic Findings

**Figure 1:**
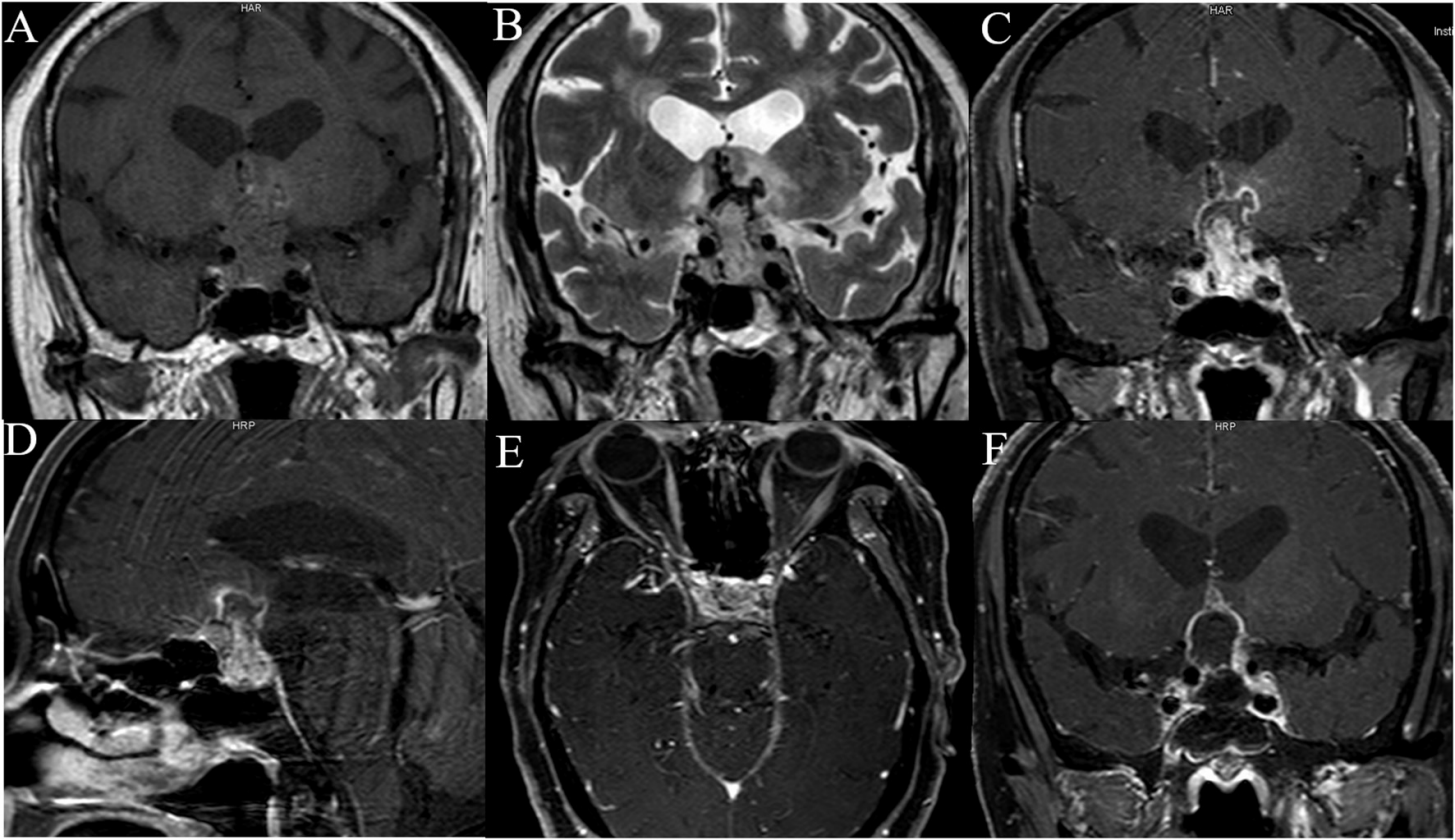
The neuroimaging of case 5. Pre-operative T1-weighted image **(A)**, T2-weighted image **(B)**, coronal **(C)**, sagittal **(D)**, axial **(E)**, T1-weighted contrast-enhanced MRI of case 5 show intrasellar heterogeneously enhanced solid and cystic mass with suprasellar extension compressing and encasing the optic chiasm, invasion of bilateral cavernous sinus. Post-operative MRI scan of the brain shows subtotal resection of the tumor **(F)**.

All patients underwent both MRI and CT scan pre-operatively. The radiological features are listed in Table 2. Five lesions were located in the sellar region. The median tumor size was 2.7 cm (range, 1.9–4.5 cm). On CT, of the five lesions, one was high dense and four were isodense, and none of the lesions had calcification. On MRI, T1-weighted imaging showed all five lesions to be isointense, and on T2-weighted imaging, three lesions were hyperintense, one isointense, and one isointense with hyperintense. Whereas, one patient experienced intratumoral hemorrhage, none showed hydrocephalus. Four of the five lesions appeared solid and one was solid and cystic. Most lesions exhibited heterogeneous enhancement and had irregular shapes (Figure 1). Peritumoral edema of the lesions that frequently extended into the cavernous sinus and also involving the surrounding brain tissue was noted.

**Table 2:** Radiological features of five adult patients with sellar region AT/RT.

**Case number**	**Maximum diameter (cm)**	**Calcification**	**Cystic component**	**Intratumoral hemorrhage**	**CT density**	**MRI**			**Peritumoral edema**	**Tumorshape**
						**T1WI**	**T2WI**	**Enhancement**		
1	4.5	No	No	No	Isodense	Isointense	Isointense	Homogeneous	No	Irregular
2	2.8	No	No	No	Isodense	Isointense	Hyperintense	Heterogeneous	No	Irregular
3	2.7	No	No	No	Isodense	Isointense	Hyperintense	Heterogeneous	No	Irregular
4	1.9	No	Yes	No	Hyperdense	Isointense	Hyperintense	Homogeneous	No	Irregular
5	2.2	No	Yes	Yes	Isodense	Isointense	Isointense with hyperintense	Heterogeneous	Yes	Irregular

### Pathological Examination

**Figure 2:**
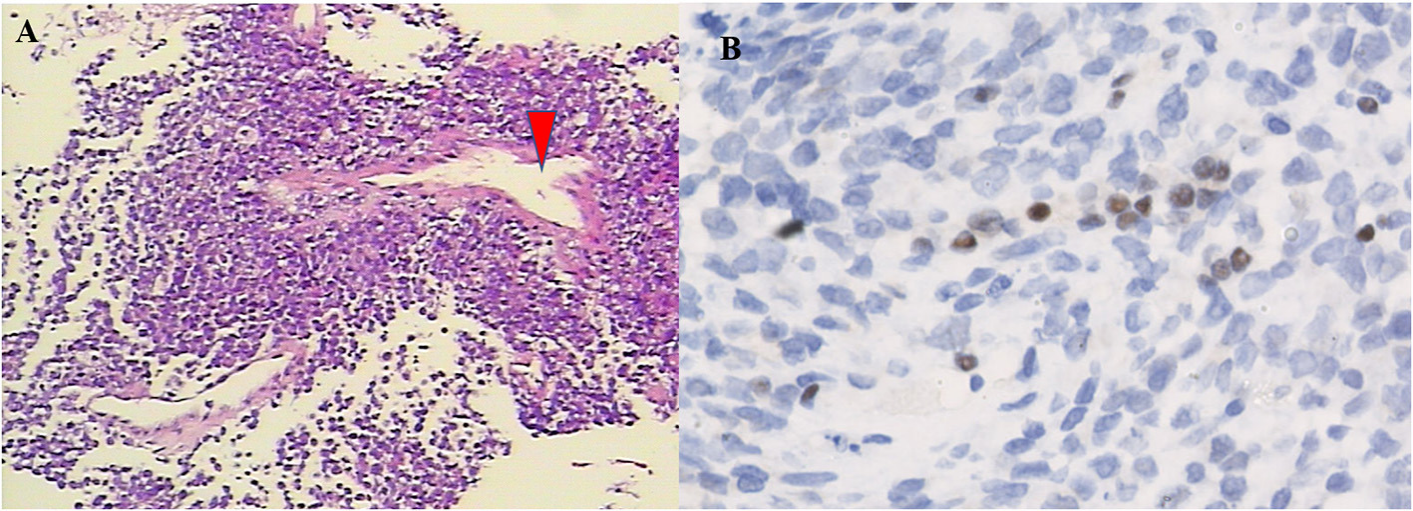
Pathological features of the tumor. On H&E staining (**A**, ×100), the tumor was composed of atypical cells with pink cytoplasm and prominent nuclei, which arranged in fascicular pattern forming a thin-walled stag-horn hemangiopericytoma-like vasculature (arrowhead). Immunohistochemistry. The tumor cells lack expression of the INI1 protein (**B**, ×400).

The pathological data are summarized in Table 3. Histologically, adult sellar region AT/RT consisted of medium-sized cells showing identifiable rhabdoid morphology and some atypical epithelioid-like spindle cells. Primitive neuroepithelial or interstitial components were also observed. Notably, three of the five lesions had hemangiopericytoma-like stag-horn vasculature (Figure 2). Immunohistochemistry showed loss of INI1, S-100, GFAP, and desmin expression in all five lesions, which were immunopositive for CD99 (66.6%; 2 of 3), CK (60.0%; 3 of 5), CD34 (33.3%; 1 of 3), EMA (50%; 2 of 4), and Syn (10%; 1 of 5). Staining for MIB-1/Ki-67 index test showed median MIB1/Ki-67 levels of 40% in all five lesions (range, 20–50%).

**Table 3:** Immunohistochemical characteristics of five adult patients with sellar region AT/RT.

**Case Number**	**INI1 deficiency**	**EMA**	**S-100**	**GFAP**	**Syn**	**CK**	**Desmin**	**CD99**	**CD34**	**MIB1/Ki-67**
1	+	+	-	-	-	-	-	-	+	20%
2	+	-	-	-	-	-	-	NA	-	50%
3	+	NA	-	NA	-	+	-	+	NA	40%
4	+	+	-	-	-	+	-	+	-	30%
5	+	-	NA	-	+	+	NA	NA	NA	50%

### Treatment and Outcomes

All five patients underwent surgical resection via endoscopic transnasal sphenoidal approach. Since the tumor exhibited blurred boundaries with the surrounding structure and invaded into the cavernous sinus and brain tissue, only one patient underwent GTR whereas the other four underwent STR. No surgery-related complications were observed during the early post-operative period. On 1-month post-operation, two patients (case 4 and case 1) had an excellent KPS score of 100 and 90, respectively. However, one patient (case 2) experienced disease progression (KPS score, 50) and two patients (case 3 and case 5) died (KPS score, 0). One patient (case 4) who survived 8 months received post-operative adjuvant radiotherapy with a total dose of 60 Gy applied in 30 fractions and local gamma knife radiation therapy once. One patient (case 1), with an OS of 4 months, received 52 Gy radiation in 28 fractions followed by six cycles of chemotherapy (cisplatin and dacarbazine). Because of the advanced age and poor medical condition of case 5, the family refused post-operative adjuvant radiotherapy and chemotherapy. Two patients (case 2 and case 3) showed rapid disease progression after resection, leading to deterioration of their condition, and therefore did not complete post-operative RT and chemotherapy course. Clinical follow-up after surgery was available for all five patients. At the last follow-up, the median RFS was 1 month (range, 0.5–2 months) and the median OS was 2 months (range, 1–8 months).

### Reported Data Review

All searches generated 235 articles in total. After carefully reading of the titles and abstracts, 188 irrelevant articles, 3 non-English, and 10 repeated articles were excluded. Thirty-four relevant reports were selected for a full-text review. Finally, the remaining 26 reports are summarized in Table 4 (7–32).

**Table 4:** Key characteristics of reported cases of adult sellar region AT/RT.

**Investigator**	**Age/sex**	**Symptoms**	**Duration of symptoms (months)**	**MRI/maximum diameter**	**Extent of resection**	**RT**	**CT**	**INI1immunohistochemistry**	**MIB1/Ki-67**	**State**	**OS (months)**
Kuge et al. (8)	32/F	Severe headache, nausea visual disturbance	NA	Enhanced mass/2.0	STR	Y	Y	Loss	NA	Dead	28
Raisanen et al. (9)	31/F	NA	NA	Enhanced mass/2.0	SR	Y	N	Loss	NA	Alive	9
	20/F	Vision loss	NA	Heterogeneous enhancement/1.6	GTR	Y	Y	Loss	NA	Dead	28
Arita et al. (10)	56/F	Headache and double vision	2	Heterogeneous enhancement/2.5	STR	Y	N	Loss	30%	Dead	23
Schneiderhan et al. (11)	61/F	Incomplete palsy of the left 6th cranial nerve	NA	Heterogeneous enhancement/NA	STR	N	N	Loss	50%	Dead	3
	57/F	Headache, double vision oculomotor nerve palsy	NA	Heterogeneous enhancement/NA	GTR	Y	Y	Loss	80%	Alive	6
Moretti et al. (12)	60/F	Frontotemporal stabbing pain, diplopia	NA	Heterogeneous enhancement/NA	STR	Y	Y	Loss	30%	Dead	30
Chou et al. (13)	43/F	Headache and diplopia	NA	Homogeneous enhancement/NA	STR	Y	NA	Loss	NA	Alive	0.5
Park et al. (14)	42/F	Visual disturbance	NA	Heterogeneous enhancement/NA	STR	Y	Y	Loss	NA	Alive	27
Shitara et al. (15)	44/F	Visual disturbance	2	Heterogeneous enhancement/NA	STR	Y	Y	Loss	85%	Dead	17
Lev et al. (16)	36/F	Headache, blurry vision	0.23	Heterogeneous enhancement/3.3	GTR	Y	Y	Loss	NA	Dead	29
Biswas et al. (17)	48/F	Visual field disturbance	0.5	NA	GTR	Y	Y	Loss	NA	Died	2
Larrán-Escandón et al. (18)	43/F	Headaches, vomiting, lower limb weakness, diplopia and ptosis	3.5	NA//2.3	STR	N	N	Loss	NA	Dead	1
Nobusawa et al. (19)	69/F	Ptosis and double vision	NA	Heterogeneous enhancement/2.8	GTR	Y	Y	Loss	60%	Alive	24
Elsayad et al. (20)	66/M	Diplopia and a headache	NA	Heterogeneous enhancement/2.2	GTR	Y	Y	Loss	NA	Alive	48
Almalki et al. (21)	36/F	Severe headache, double vision and vomiting	3	Heterogeneous enhancement/NA	STR	Y	Y	Loss	NA	Alive	36
Dardis et al. (22)	35/M	Blurred vision	3	Heterogeneous enhancement/NA	GTR	Y	Y	Loss	NA	Alive	30
Nakata et al. (7)	26/F	NA	NA	NA	STR	Y	Y	Loss	30%	Dead	33
	21/F	NA	NA	NA	STR	Y	Y	Loss	26%	Dead	35
Barresi et al. (23)	59/F	Headache, diplopia, and visual disturbance	NA	Heterogeneous enhancement/2.3	STR	Y	N	Loss	70%	Dead	2
Nishikawa et al. (24)	42/F	Severe headache, vertigo, visual disturbance	NA	Homogeneous enhancement/2.0	STR	Y	Y	Loss	30%	Dead	11
Johann et al. (25)	66/M	NA	NA	NA	NA	NA	NA	Loss	NA	Alive	54
	20/F	NA	NA	NA	NA	Y	Y	Loss	NA	Dead	120
	48/F	NA	NA	NA	NA	NA	NA	Loss	NA	Alive	4
Paolini et al. (26)	65/F	NA	NA	NA	STR	Y	Y	Loss	NA	Dead	23
	47/F	NA	NA	NA	STR	Y	Y	Loss	NA	Alive	62
	31/F	NA	NA	Heterogeneous enhancement/NA	STR	N	N	Loss	NA	Dead	2
	36/F	NA	NA	NA	STR	Y	Y	Loss	NA	Alive	22
Voisin et al. (27)	51/F	Visual disturbance and peripheral vision loss	5	Heterogeneous enhancement/NA	STR	Y	Y	Loss	7%	Alive	9
Asmaro et al. (28)	62/F	Headache, nausea, double vision	Several months	Heterogeneous enhancement/NA	STR	N	N	Loss	NA	Dead	2
Ahmad et al. (29)	33/F	Intractable global headache, diplopia, III and VI nerve palsies	0.23	Homogeneous enhancement/1.5	STR	Y	Y	Loss	30%	Alive	36
Siddiqui et al. (30)	55/F	Vision disturbance, headache	0.23	Heterogeneous enhancement/5.7	GTR	NA	NA	Loss	Elevated	Dead	1.5
Bokhari et al. (31)	40/F	Severe headache decrease vision	Few days	Heterogeneous enhancement/2.9	STR	Y	Y	Loss	NA	Dead	2
Oraibi et al. (32)	70/F	Severe headache	NA	NA	STR	Y	Y	Loss	60%	Dead	6
Present case 1	43/F	Headache, diplopia, ptosis	0.23	Homogeneous enhancement/4.5	STR	Y	Y	Loss	20%	Dead	4
Present case 2	52/F	Headache, numbness in limbs	0.5	Heterogeneous enhancement/2.8	STR	N	N	Loss	50%	Dead	2
Present case 3	50/F	Headache, visual disturbance, galactorrhea, numbness in limbs	2	Heterogeneous enhancement/2.7	GTR	N	N	Loss	40%	Dead	1
Present case 4	29/F	Headache	4	Homogeneous enhancement/1.9	STR	Y	N	Loss	30%	Dead	8
Present case 5	80/F	Headache, visual disturbance, ptosis	0.5	Heterogeneous enhancement/2.2	STR	N	N	Loss	50%	Dead	1

*NA, not available; F, female; M, male; STR, subtotal resection; GRT, gross total resection; CT, chemotherapy; RT, radiotherapy; SR, surgery; OS, overall survival*.

**Figure 3:**
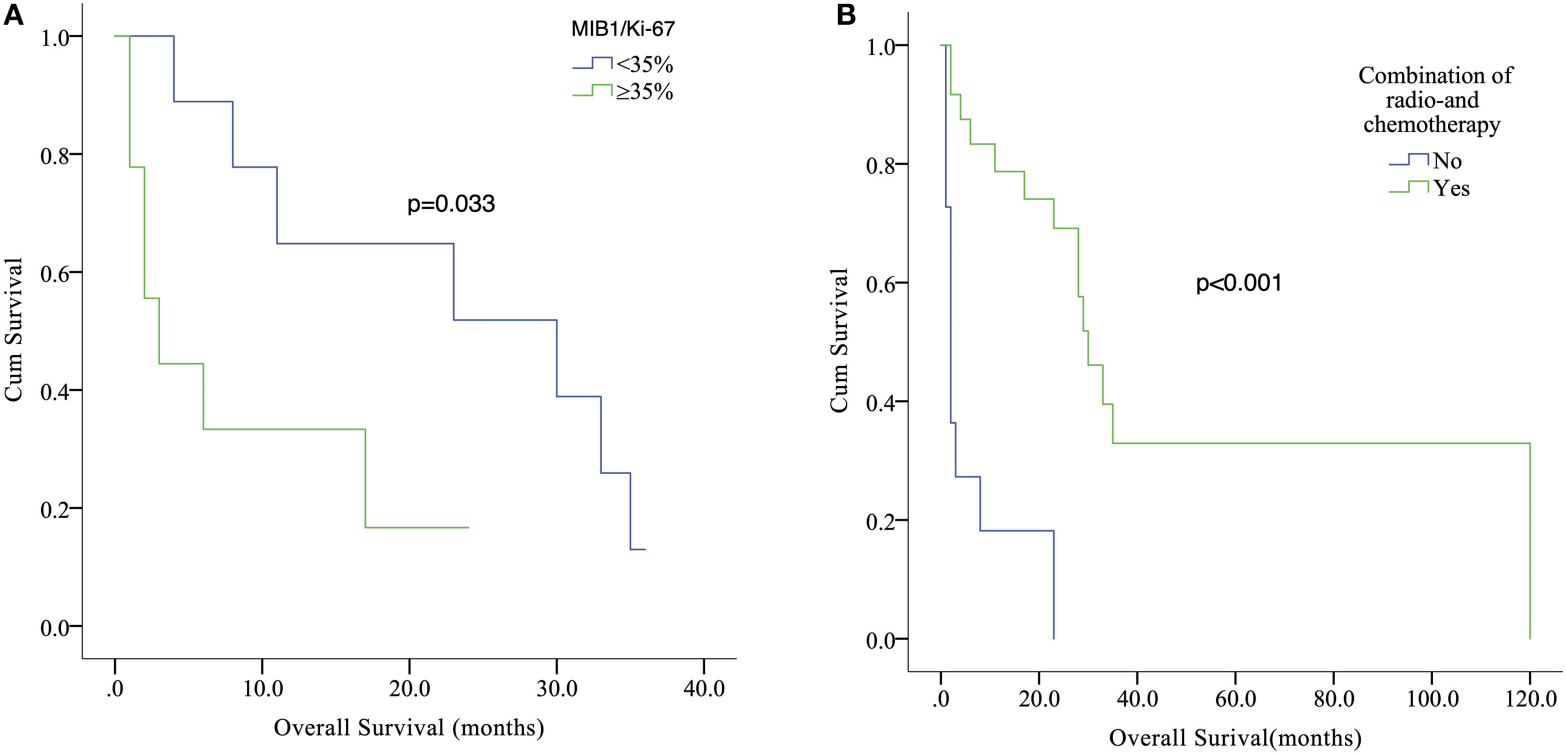
Kaplan–Meier analysis showed that patients with high (≥35%) MIB-1/Ki67 index value had a significantly shorter OS compared with those with low (<35%) index value (*p* = 0.033) **(A)**. Relationship between post-operative combination radio- and chemotherapy (Yes or No) and survival outcome (*p* < 0.001) **(B)**.

Since the initial description of adult sellar AT/RT, 39 cases, including our case series, constitute the current main body of reported data on adult sellar AT/RT. The estimated median OS was 23 months (range, 1–120 months) with a 1-year survival estimate of 59.7%. The mean age was 46.3 ± 15.1 years. Of the 39 patients, 36 (94.9%) were female and 3 (7.7%) were male. The symptoms were reported in 29 patients, and 20 patients (92.3%) suffered from headache and visual disturbance. The characteristics of contrast enhancement data were available for 26 patients, and the heterogeneous enhancement was observed in 21 of these patients (72.4%). The characteristics of contrast enhancement (*p* = 0.621) did not have a significant effect on the OS in Kaplan–Meier analysis. In addition, the median value of MIB1/Ki-67 was 35% (range, 7–85%) in 18 patients, and those patients were classified into two groups by the median value of MIB1/Ki-67. Kaplan–Meier analysis showed that patients with high (≥35%) MIB-1/Ki67 index value had a significantly shorter OS compared with those with low (<35%) index value (*p* = 0.033; Figure 3A). Furthermore, of the 39 patients, 36 underwent surgical resection including 26 STR, 9 GTR, and one with an unknown extent of resection, while the treatment information for the remaining 3 patients were unavailable. The median OS for patients with GTR was 28 and 17 months for patients with STR. However, the Kaplan–Meier analysis results did not show any significant difference between extent of surgical resection and OS (*p* = 0.501). The data of post-operative adjuvant therapy were available in 35 patients. Twenty-four received combination radio- and chemotherapy; four received radiation therapy alone after resection and none of the cases received chemotherapy alone. The Kaplan–Meier analysis demonstrated that patients who received post-operative combination radio- and chemotherapy had a significantly longer OS than that of those who did not (*p* < 0.001; Figure 3B). In addition, the role of radiotherapy toward adult sellar AT/RT could not be affirmatively concluded because of the small sample size.

## Discussion

### Clinical Characteristics

Adult sellar region AT/RT is an extremely rare and fast-growing neoplasm of the CNS. To the best of our knowledge, only 34 cases of adult sellar region AT/RT were previously reported in English-language journals in the form of case reports or retrospective case series. A literature search of all previous reports of adult sellar region AT/RT are summarized in Table 4 (7–32).

Thus far, only three cases of adult male patients with sellar region AT/RT have been described (20, 22, 25), indicating a female gender preponderance, which was also reflected in our case series. This suggests that women are susceptible to sellar region AT/RT, but further studies are warranted to reach a definitive conclusion. Interestingly, in the pediatric AT/RT population, there is a higher incidence in males (5, 33). Furthermore, pediatric AT/RT occurs chiefly <3 years old (34). The mean age was 46.3 ± 15.1 years in the 39 cases discussed here. The average symptom duration of adult sellar region AT/RT in our case series was 1.6 months (range, 2 weeks−8 months), which was consistent with previously reported studies (range, 1 week−5 months) (10, 15–18, 21, 22, 27). Because other sellar region diseases may exhibit similar symptoms, the clinical manifestations of adult sellar region AT/RT are likely non-specific, and mainly depend on the tumor size. Indeed, the most frequently reported symptoms were headache and visual disturbance (1–3, 7, 9, 14, 15, 21–26, 28–31, 33–38). Remarkably, headache in the five cases was usually severe and unbearable, characterized by whole-brain distending pain that was not mitigated by mild analgesics or weak opioids.

Pre-surgery levels of serum pituitary hormone levels are non-specific for differential diagnosis of adult sellar region AT/RT because pituitary hormone deficiency is also seen in large non-functioning pituitary adenoma (35). There are contradicting reports of serum pituitary hormone levels within the normal range before surgery (12, 23, 24) or diminished levels in adult sellar region AT/RT patients (16, 21). Accordingly, in our study, pituitary hormone deficiency was found in four patients, and anterior pituitary function was normal in one patient. The cause of hypopituitarism may be related to the lesion compression or invasion of the normal pituitary gland.

### Radiologic Findings

Radiologic features are crucial for pre-operative diagnosis of adult sellar region AT/RT and CT and MRI are the common modalities of choice for diagnosing these tumors. CT scan of the adult AT/RT usually show either isodense or, rarely, hyperdense lesions (12, 18, 28), and accordingly, 80% of adult sellar region AT/RTs in our case series were isodense, and 20% hyperdense. Rare hyperdense lesions of sellar region AT/RT usually indicate intratumor hemorrhage, which was confirmed in the present study and Asmaro et al. (28) Hyperdense sellar and suprasellar mass also should be differentiated from craniopharyngioma or pituitary macroadenoma by CT imaging. It was isointense on T1-weighted, hyperintense or isointense on T2-weighted images (14, 16, 21). The lack of calcification in our five adult sellar region AT/RT cases and the accompanying features of the lesions, namely, irregular shapes, and involving brain tissue, have not been reported in the literature. On MRI, the lesions extended into the cavernous sinus, compressing and displacing the optic chiasm, and involved the surrounding brain tissue. In addition, the majority of sellar region AT/RT showed heterogeneous enhancements, and characteristics of contrast enhancement did not have a significant effect on the OS. Noticeably, in the sellar region, pituitary tumors that are relatively regular and hourglass in shape often exhibit homogeneous enhancements. Furthermore, the slow growth of pituitary tumors relative to the sellar region AT/RT. Thus far, none of pediatric sellar AT/RT was reported (27). Therefore, the radiological features of the pediatric sellar AT/RT were unclear.

### Pathological Findings

The morphological and immunohistochemical features of the tumor predominantly dictate diagnosis of AT/RT. Intraoperatively, soft-textured tumors colored gray-white or gray-red with abundant blood supply and blurred boundaries with surrounding structure that had invaded the cavernous sinus and clivus were observed in our case series. Histologically, the AT/RT tumors were densely packed with rhabdoid, neuroepithelial, and epithelial cells that were teratoid in nature (36). In our study, most lesions in the adult sellar region AT/RT had hemangiopericytoma-like stag-horn vasculature on histopathologic examination, which may differ from that of non-sellar region AT/RT (7, 19). Immunohistochemical findings of cellular markers in the adult sellar region AT/RT of our cases were in concordance to that observed previously (9, 37). The lack of expression of S-100, GFAP, and desmin in adult sellar region AT/RT in our cases is neither universal nor specific because these markers can be positive in some adult sellar region AT/RTs (11, 12, 16). Noticeably, the loss of INI1/SMARCB1/SMARCA4/BRG1 protein expression has been reported as a main feature of AT/RT in 34 cases examined in the literature (3, 39), which was similar to the five cases in our study.

The robust expression of MIB1/Ki-67 may imply the highly proliferative nature of an aggressive tumor. The median value of MIB1/Ki-67 was 35% in our pooled analysis (range, 7–85%). The Kaplan–Meier analysis showed that patients with high (≥35%) MIB-1/Ki67 index value had a significantly shorter OS compared with those with low (<35%) index value (*p* = 0.033). It was our novel finding that high levels (≥35%) of MIB1/Ki-67 in the adult sellar region AT/RT may indicate aggressive feature.

### Treatment and Outcomes

Because of the rareness of adult sellar region AT/RT, a consistent treatment protocol has not yet been established. However, a multimodal approach including surgical resection, radiation, and chemotherapy has been applied for the treatment of adult sellar region AT/RT. Although surgical resection is the main treatment, the goal of gross total resection is difficult to achieve because of the unclear boundaries, abundant blood supply, and invasion of the tumor into the cavernous sinus and clivus. Of the 39 cases, 36 underwent surgical resection including 26 STR, 9 GTR, and one with an unknown extent of resection (9), whereas the treatment information for the remaining three patients were unavailable (25). Furthermore, the extent of resection on OS remains unclear. Whereas, some studies noted a favorable outcome with GTR (15, 38, 40), others found no difference in survival between those who received GTR and STR (5, 41). We found that the median OS for patients with GTR was 28 months and 17 months for patients with STR. However, the Kaplan–Meier analysis results did not show any significant difference between extent of surgical resection and OS. Nevertheless, considering that adult sellar region AT/RT is aggressive in nature and fast-growing, maximal safe resection may be the optimal strategy, although the prognostic impact of extent of resection on sellar region AT/RT remains controversial. For pediatric patients with AT/RT, some studies have reported that GTR was likely beneficial when compared with STR (33, 42). However, other studies found no significant difference in the survival based on the extent of resection (35, 43).

Although post-operative chemotherapy using ifosfamide, carboplatin, and etoposide is an important supplementary therapy for AT/RT, and doxorubicin, methotrexate, and temozolomide have been explored for adult sellar region AT/RT (7, 16, 17, 19), the best chemotherapeutic regimen for adult AT/RT remains unclear (44, 45). Some of these agents can favorably penetrate into the intraparenchymal tumor and cerebrospinal fluid (44, 45). Because none of the 34 previous cases and in our case series received chemotherapy alone after resection, the responsiveness of adult sellar region AT/RT to chemotherapy cannot be assessed and thus, remains to be proven. However, chemotherapy has shown benefits; Hilden et al. (46) showed that post-operative chemotherapy achieved complete (6 patients) or partial (6 patients) response in 12 of 22 pediatric AT/RT cases.

The benefits of radiotherapy have been explored to treat and control both primary AT/RT and micro-metastatic disease (47, 48). Buscariollo et al. (35) reported that the median survival of 144 patients with AT/RT who received radiotherapy improved from 6 to 34 months. Another study documented that post-operative local radiotherapy for AT/RT with 54 Gy in 1.8 Gy daily fractions increased potential survival (20). Besides, several factors such as dose, technique, and timing can affect the outcomes from radiotherapy. Elsayed et al. (20) indicated that focal radiation doses ranging from 30 to 60 Gy can improve local control of AT/RT. Chen et al. (49) showed an increased disease-free survival in AT/RT from a total dose of more than 50 Gy and found that any delay in initiation of radiation therapy had an adverse effect on prognosis. Similarly, Tekautz et al. (48) also revealed that radiotherapy delivered early or late after surgical resection was associated with survival. Three cases from our literature review of 34 cases of adult sellar region AT/RT, and one patient (case 4) in our case series, who had an OS of 8 months, received post-operative radiotherapy alone. Because of these small sample sizes, we cannot further evaluate the OS benefits of radiotherapy alone in adult sellar region AT/RT.

The role of combination of radio- and chemotherapies among adult sellar region AT/RT patients remains to be determined (14, 26). We attempted to explore the effect of post-operative combination of radio- and chemotherapy on outcomes in adult sellar region AT/RT patients. In our pooled analysis, the data of post-operative adjuvant therapy was available in 35 patients, and 24 received combination radio- and chemotherapy. The Kaplan–Meier analysis demonstrated that patients who received post-operative combination radio- and chemotherapy had a significantly longer OS than that of those who did not (*p* < 0.001). Our results showed that combination of radio- and chemotherapy may be beneficial for adult sellar region AT/RT patients after surgical resection. In addition, further clinical studies may be needed to verify its efficacy. When it comes to the combination of radio- and chemotherapy for pediatric AT/RT, some of studies have demonstrated that the post-operative combination of radio- and chemotherapy was associated with better outcomes (33, 48).

Despite efforts to develop multimodal treatments, the overall prognosis of adult sellar region AT/RT remains poor. We found that adult patients with sellar region AT/RT had a median OS of 23 months (range, 1–120 months) in the 39 cases discussed here with a 1-year survival estimate of 59.7%, which was significantly longer than the median OS of 11.1 months among children with AT/RT (50). To improve OS, the novel treatment approaches are urgently needed for adult and pediatric AT/RT.

### Study Limitations

This study is not without limitations. First of all, it was limited by the retrospective nature and small sample size. Some of the results were based on pooled data from various cases or case series; therefore, heterogeneities in patient populations and treatment regimens should be kept in mind when interpreting the results. In addition, the information about MRI spine was lacking. As a result, the rarity of the disease limits additional statistical analyses, and finally, data on immunohistochemical staining were incomplete. Even though our study was the largest retrospective study on adult sellar region AT/RT until now, which can be helpful in the comprehensive understanding and management of adult sellar region AT/RT. Further prospective multicenter studies with larger sample sizes are warranted to support our results and identify the best treatment strategy for adult patients with sellar region AT/RT.

## Conclusions

Adult sellar region AT/RT is a rapidly growing rare tumor in the CNS with a poor prognosis. Headache, usually severe and unbearable, is the main symptom. The disease shows a striking female predominance, which is its most prominent feature. Adult sellar region AT/RT usually exhibits irregular shape and heterogeneous enhancement and frequently extends into the cavernous sinus, involving even the surrounding brain tissue on MRI. The CT scan usually shows an isodense mass and rarely calcification. It was our novel finding that high levels of MIB1/Ki-67 may indicate aggressive feature. When an adult female patient has these features, it is recommended to include adult AT/RT in differential diagnosis of unclear malignant sellar lesions. Maximal safe resection followed by adjuvant radiotherapy combined with chemotherapy may be the optimal therapeutic strategy for adult sellar region AT/RT, and further clinical studies may be needed to verify its efficacy.

## Data Availability Statement

The raw data supporting the conclusions of this article will be made available by the authors, without undue reservation.

## Ethics Statement

The studies involving human participants were reviewed and approved by Sichuan University. The patients/participants provided their written informed consent to participate in this study. Written informed consent was obtained from the individual(s) for the publication of any potentially identifiable images or data included in this article.

## Author Contributions

FL and ShucF: data curation. FL and XT: formal analysis. ShuaF and ShucF: methodology. FL: writing—original draft. LZ: writing—review and editing. All authors contributed to the article and approved the submitted version.

## Conflict of Interest

The authors declare that the research was conducted in the absence of any commercial or financial relationships that could be construed as a potential conflict of interest.

## Abbreviations

AT/RT: Atypical teratoid/rhabdoid tumor
CNS: Central nervous system
GTR: Gross total resection
KPS: Karnofsky performance scale
OS: Overall survival
RFS: Recurrence-free survival
STR: Subtotal resection.
